# Decomposition of Organic Carbon in Fine Soil Particles Is Likely More Sensitive to Warming than in Coarse Particles: An Incubation Study with Temperate Grassland and Forest Soils in Northern China

**DOI:** 10.1371/journal.pone.0095348

**Published:** 2014-04-15

**Authors:** Fan Ding, Yao Huang, Wenjuan Sun, Guangfu Jiang, Yue Chen

**Affiliations:** 1 State Key Laboratory of Vegetation and Environmental Change, Institute of Botany, Chinese Academy of Sciences, Beijing, China; 2 University of Chinese Academy of Sciences, Beijing, China; Tennessee State University, United States of America

## Abstract

It is widely recognized that global warming promotes soil organic carbon (SOC) decomposition, and soils thus emit more CO_2_ into the atmosphere because of the warming; however, the response of SOC decomposition to this warming in different soil textures is unclear. This lack of knowledge limits our projection of SOC turnover and CO_2_ emission from soils after future warming. To investigate the CO_2_ emission from soils with different textures, we conducted a 107-day incubation experiment. The soils were sampled from temperate forest and grassland in northern China. The incubation was conducted over three short-term cycles of changing temperature from 5°C to 30°C, with an interval of 5°C. Our results indicated that CO_2_ emissions from sand (>50 µm), silt (2–50 µm), and clay (<2 µm) particles increased exponentially with increasing temperature. The sand fractions emitted more CO_2_ (CO_2_-C per unit fraction-C) than the silt and clay fractions in both forest and grassland soils. The temperature sensitivity of the CO_2_ emission from soil particles, which is expressed as Q_10_, decreased in the order clay>silt>sand. Our study also found that nitrogen availability in the soil facilitated the temperature dependence of SOC decomposition. A further analysis of the incubation data indicated a power-law decrease of Q_10_ with increasing temperature. Our results suggested that the decomposition of organic carbon in fine-textured soils that are rich in clay or silt could be more sensitive to warming than those in coarse sandy soils and that SOC might be more vulnerable in boreal and temperate regions than in subtropical and tropical regions under future warming.

## Introduction

As the largest terrestrial carbon pool, soil plays a vital role in the global carbon cycle [1]. A small change in the magnitude of soil carbon stocks could result in a large impact on the atmospheric enrichment of CO_2_ [2]. Soil organic carbon (SOC) loss via soil heterotrophic respiration (i.e., SOC decomposition) is one of the largest components of global CO_2_ flux from the terrestrial ecosystem to the atmosphere [3], [4]. It is widely recognized that temperature has a profound influence on SOC decomposition [5] and that soils are expected to release CO_2_ at faster rates with rising temperature [3]. Therefore, understanding the temperature sensitivity of SOC decomposition is critical for predicting changes in global soil respiration, changes in soil carbon stocks to future warming, and especially, the feedback of soils on climate change [2], [6]–[8].

The temperature sensitivity of SOC decomposition, which is commonly expressed as Q_10_, refers to the change in decomposition rate with a 10°C increase in temperature. A recent study constructed a database of worldwide soil respiration observations matched with high-resolution historical climate data and estimated that global soil respiration had a Q_10_ of 1.5 [3]. Nevertheless, soil respiration experiments have unanimously indicated that Q_10_ is spatially heterogeneous on a global scale [9], [10]. Thus, to accurately predict changes in global soil carbon stocks to future warming, knowing which factors determine the temperature dependence of SOC decomposition on large scales is critical [6]. It is known that the Q_10_ of soil heterotrophic respiration varies with temperature [5], [11], soil moisture [12], [13], and the quantity and quality of SOC [14], [15]. However, it is not clear whether SOC decomposition has different warming responses in different soil textures. It is most likely so because the SOC stock in fine-textured soils is less vulnerable to disturbances, e.g., tillage, than in coarse-textured soils [16], [17].

Such different vulnerabilities are assumed to result from fine-textured soils having greater fractions of silt-sized or clay-sized particles, whereas coarse-textured soils have more sand-sized particles; these primary particles contain different mineral compositions and provide various affinities to organic matter [18]. Clay and silt particles (e.g., sesquioxides and layer silicates) provide large specific surface areas and numerous reactive sites at which SOC can be sorbed by strong ligand exchange and polyvalent cation bridges, whereas the sand particles, which is dominated by quartz particles, exhibits only weak bonding affinities to SOC [19], [20]. The bonding of SOC to minerals is recognized to prevent decomposition [21], [22] and may therefore decrease the temperature sensitivity of decomposition [23]. Thus, the decomposition of organic carbon associated with sand, silt, and clay fractions likely respond to warming differently. The temperature sensitivity of SOC decomposition from different particle size fractions is intimately linked to the response of CO_2_ emission from different soil textures to future warming and the emission's feedback on climate change.

Different soil particle size fractions possess different sizes of carbon pools [20]. Clay and silt have large specific surface areas and can sorb abundant SOC [24]. For example, in temperate arable soils, 50–75% of the total SOC is associated with clay, whereas silt accounts for 20–40% and sand accounts for less than 10% [18]. Additionally, the SOC associated with the clay and silt fractions is assumed to be stable relative to that associated with the sand fraction [21]. In this context, the long-term stabilization of soil carbon pools under climate warming mainly depends on the warming response of the carbon that is associated with the clay and silt fractions.

However, to our knowledge, there were few studies who explored difference in the warming responses of organic carbon associated with these primary particles. In this study, we conducted a 107-day incubation experiment to investigate the temperature sensitivity of SOC decomposition in sand (>50 µm), silt (2–50 µm), and clay (<2 µm) particles from grassland and forest soils. This study aims to answer the following questions: 1) Is there a difference in the Q_10_ of SOC decomposition among different size soil particles and between the two different ecosystems? 2) Which factors determine the Q_10_? A further objective is to better understand the features of CO_2_ emission and their future trends with global warming for different textured soils.

## Materials and Methods

### Sites and sampling

Soil samples were collected from two sites at the typical steppe grassland and temperate forest ecosystems in northern China. The grassland site was at the Duolun Restoration Ecology Experimentation and Demonstration Station (42°02′N, 116°17′E, 1324 m a.s.l.) and the forest site was at the Beijing Forest Ecosystem Research Station (39°58′N, 115°26′E, 1252 m a.s.l.) at the Institute of Botany of the Chinese Academy of Sciences. The grassland is permanent grassland situated in the temperate continental semi-arid climate zone. The dominant species are *Stipa baicalensis*, *Thalictrum petaloideum*, and *Artemisia frigida*. The mean annual temperature (MAT) is 1.6°C, and the mean annual precipitation (MAP) is 386 mm, with 100 frost-free days. The soil is sandy Haplic Calcisols (FAO) composed of 61.2% sand, 23.0% silt, and 15.8% clay, and the pH is 7.0. The forest is an artificial forest (with age >40 years) with a dominant *pinus tabuliformis Carrière* specie and the climate is warm temperate humid monsoon climate zone. The MAT and MAP are 5°C and 650 mm, respectively, with 160 frost-free days. The soil is a clay loam that belongs to Eutric Cambisol (FAO) and is composed of 41.0% sand, 42.5% silt, and 16.5% clay, and the pH is 7.0.

At each site, 40 cores of mineral soils at 0–20 cm depths were randomly collected using an auger with 4 cm in diameter and were composited into one soil sample. A small fraction of the soils (approximately 1 kg) was immediately placed in a sterile plastic bag on ice when transported to the laboratory and stored at 4°C for inoculum preparation. The remaining soils were placed into cloth bags and air-dried at room temperature. The dried soils were finely crumbed and sieved through a 2-mm mesh to remove visibly identifiable plant debris and gravel. Fine roots were removed through static electricity sorption.

### Particle-size fractionation

Twenty grams of dried soil were dispersed in 100 ml of deionized water by the ultrasonic probe of a JY92-II Ultrasonic Homogenizer (6 mm diameter probe, 300 W). The ultrasonic time was chosen as 3 min and 8.5 min for grassland and forest soil, respectively, after calibrating against conventional soil texture analyses [25]. The dispersed suspensions were separated into three size fractions: sand (>50 µm), silt (2–50 µm), and clay (<2 µm). The sand fraction was separated using distilled water to wash all of the fine particles in the soil suspension through a 50-µm sieve until the elutriate solution that exited the sieve was clear. The elutriate was divided into silt and clay fractions by repeated centrifugation using an Avanti J-25 Beckman Centrifuge [26]. The clay fraction was concentrated from the large volumes of suspensions by flocculation with CaCl_2_ and centrifugation [27]. The three size fractions were oven-dried at 40°C [28], gently crushed, weighed, and then stored for incubation and future analysis.

### Laboratory incubation

**Figure 1:**
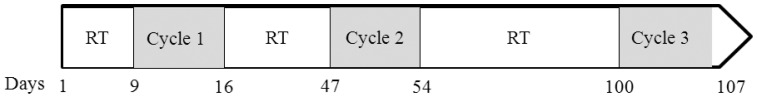
Schematic diagram of the time sequence for the three temperature-change cycles. RT signifies that the soil samples were incubated at room temperature.

For each soil particle, a 10-g dried sample was placed into a 150-ml brown flask. Then, 2 ml of inoculum were added to each sample, and the inoculum solution was prepared by shaking 100 g of fresh soil with 1000 ml of deionized water and leaving to stand overnight [28]. To improve soil aeration and make the inoculum disperse uniformly, the sample plus inoculum was loosened, stirred, and leveled slightly. The soil moisture was adjusted to 60% water-filled pore space (WFPS) [29]. The incubation lasted 107 days (Figure 1). The soil samples were incubated at room temperature (ca. 20°C) when there was no measurement. The samples were regularly corrected for water loss by adding deionized water. Four replicates were conducted in the experiment. Empty flasks were set as blank. The volume of head space (flask volume minus soil solid) was accurately measured after complementing the experiment by the method of water replacement [30].

Following Fang et al. [31] and Xu et al. [32], soil respiration rates under changing temperature were measured in sequence. Three temperature-change cycles were started at the 9^th^, 47^th^, and 100^th^ days after the beginning of the incubation, and each cycle lasted 8 days (Figure 1). The first temperature-change cycle began after 8 days of incubation to stabilize the microbial activity to avoid an undesired microbial peak [33]. Within each cycle, the temperature was continuously increased from 5°C to 30°C in intervals of 5°C and then gradually decreased from 30°C to 5°C with the same interval. After each temperature was changed to the next, the incubation flasks were kept open for 3 hours to allow the soil samples to adapt to the changed temperature [30]; the flasks were then sealed using butyl rubber septa and flushed with ambient air for one minute using a pump with an “inlet-outlet” needle inserted through the septa to produce the same initial gas conditions among all flasks. After specific times of enclosure (20, 15, 9, 6, 4, and 3 hours for 5, 10, 15, 20, 25, and 30°C, respectively), 5 ml of gas was taken through a gas-tight injection syringe from the headspace of the flask. The CO_2_ concentrations were analyzed with a gas chromatograph (Agilent 7890A, USA).

### Determination of soil physicochemical properties

The soil texture was measured using the hydrometer method [34]. The SOC and soil total nitrogen (STN) concentrations were determined using the K_2_Cr_2_O_7_-H_2_SO_4_ wet oxidation method [35] and the Kjeldahl method [36], respectively. The microbial biomass carbon (MBC) and microbial biomass nitrogen (MBN) in the soil samples at the end of incubation were measured using chloroform fumigation-extraction (K_2_SO_4_) [37] with a Multi N/C 3100-TOC/TN Analyzer (Analytik Jena, Germany). The K_2_SO_4_-extractable organic carbon and nitrogen from the non-fumigated soil samples were considered as the dissolved organic carbon (DOC) and total dissolved nitrogen (TDN) [38], [39].

### Calculations and statistical analysis

Similar to Lang et al. [40], the CO_2_ emission rate was calculated using the following equation:
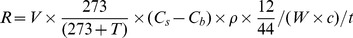
(1)where *R* is the CO_2_ emission rate (µg CO_2_-C g^−1^ OC h^−1^); *V* is the flask headspace volume (L); *T* is the incubation temperature (°C); *C_s_* and *C_b_* are the CO_2_ concentrations in the sample and blank flasks (ppm), respectively; *ρ* is the standard state CO_2_ density (g L^−1^); *W* is the dry weight of the soil particles (kg); *c* is the SOC concentration of the soil particles (g kg^−1^); and *t* is the time enclosed (h). The final CO_2_ emission rate at a given temperature was calculated as the average of the values between the increasing temperature and decreasing temperature periods [31], [32]. To illustrate the temperature sensitivity of CO_2_ emission from soil particles, we used two methods to calculate Q_10_. One method involves the commonly used first-order exponential model:
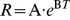
(2)where *R* is the CO_2_ emission rate (µg CO_2_-C g^−1^ SOC h^−1^), *T* is the temperature (°C), and A and B are model parameters that are to be determined. The parameter A is a carbon quality index, which refers to the availability and lability of the carbon substrates [15], [41]. The Q_10_ value was then computed as following:
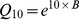
(3)


The Q_10_ calculated by Eqn. 3 was a constant value in temperature range of 5–30°C. To examine the Q_10_ variation with temperature, the Q_10_ values were also calculated separately for each temperature interval of 5 degrees (e.g., 5–10°C) and 10 degrees (e.g., 5–15°C) based on the following equation [11]:
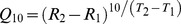
(4)where *R*_2_ and *R*_1_ are the CO_2_ emission rates (µg CO_2_-C g^−1^ SOC h^−1^) measured at temperatures *T*_2_ (high temperature) and *T*_1_ (low temperature), respectively.

All statistical analysis was conducted using SPSS 13.0 (SPSS Inc., 2004). The means were compared using the Duncan's multiple range test (DMRT).

## Results

### Physicochemical characteristics of soils

The SOC and STN concentrations of the bulk soil were very similar between the grassland and forest (Table 1). The organic carbon concentrations in grassland and forest soil particles both decreased with increasing particle size, from 55 g kg^−1^ and 73 g kg^−1^ in the clay particle (<2 µm) to 9 and 13 g kg^−1^ in the sand particles (>50 µm), respectively. Similar trends were observed for the total nitrogen concentrations. The C:N ratios were similar between the clay and silt particles, which were lower than those of the bulk soil and the sand particles. The total percentages of the carbon masses associated with clay and silt particles in the total SOC were more than 60% for both grassland and forest soils, and the percentages of their nitrogen masses in the total STN were both approximately 75%.

**Table 1:** Physicochemical composition of the soils.

Land uses and particle size fractions	Fraction a (%)	Organic carbon		Total nitrogen		C:N ratio
		Concentration (g kg^−1^)	Share of the total (%)	Concentration (g kg^−1^)	Share of the total (%)	
Grassland						
Clay (<2 µm)	11.2 (1.6)	55.3	28.5	5.8	31.9	9.5
Silt (2–50 µm)	28.0 (1.3)	30.9	40.0	3.1	43.2	9.9
Sand (>50 µm)	57.4 (1.0)	8.9	23.6	0.7	18.7	13.4
Bulk soil		21.7		2.0		10.7
Recovery (%)	96.6 (1.1)		92.1		93.9	
Forest						
Clay (<2 µm)	18.1 (2.8)	72.6	44.6	6.1	51.2	12.0
Silt (2–50 µm)	35.1 (2.7)	17.4	20.8	1.5	24.2	11.8
Sand (>50 µm)	43.9 (1.2)	13.0	19.4	0.7	14.1	18.9
Bulk soil		29.5		2.1		13.8
Recovery (%)	97.1 (1.0)		84.8		89.5	

a Values in the parentheses represent SD.

### Relationship between soil organic carbon decomposition rate and temperature

**Figure 2:**
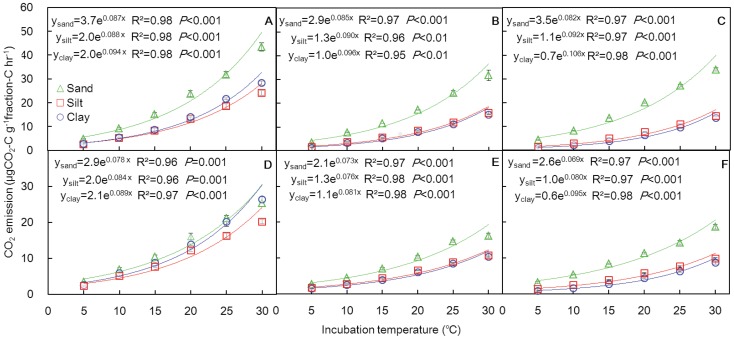
Relationship between CO2 emission rates from particle-size fractions and incubation temperature for grassland (A, B, C) and forest soils (D, E, F). The panels from left to right represent temperature-change cycles in the order 1, 2, 3. The vertical bars denote the standard deviations (n = 4).

The SOC decomposition rate from soil particle size fractions, which is expressed as the CO_2_-C emission rate per unit of fraction-C, had an exponential relationship with the incubation temperature in all measurements (Figure 2). The sand fractions, which had a lower organic carbon content (Table 1), exhibited greater SOC decomposition rates than the silt and clay fractions, which latter two were similar to one another. The decomposition rates in all the soil fractions decreased continuously as incubation time progressed (from an earlier temperature-change cycle to a later one), except for the sand fraction from the second to the third cycle. As a whole, the decomposition rates from grassland soil particles were greater than those from the corresponding forest soil particles under same condition (Figure 2).

The parameter A in the fitted equation (Eqn. 2) for the relationship between the decomposition rate and temperature is an index for carbon availability and lability. The value of parameter A was greater for the sand fraction than for the silt and clay fractions in both the grassland and forest soils (Figure 2). Furthermore, the values of parameter A were approximately equal between the two land uses for the clay and silt fractions during each temperature change cycle but were greater in the grassland than in the forest for the sand fraction (Figure 2).

### Temperature sensitivity of soil organic carbon decomposition for different soil particle-size fractions

The Q_10_ values calculated using the exponential model (Eqn. 3) were significantly different among the soil particle size fractions during each temperature-change cycle; the clay fraction exhibited the greatest value, followed by the silt and sand fractions (Table 2). The calculated mean Q_10_ values of the three cycles were 2.68±0.16 and 2.43±0.16 for the clay fraction, 2.45±0.06 and 2.22±0.10 for the silt, and 2.33±0.08 and 2.09±0.09 for the sand, in the grassland and forest soils, respectively. The mean Q_10_ values of the grassland soil particles were significantly greater than those that of the corresponding forest soil particles (Table 2). As the incubation time progressed (from an earlier temperature-change cycle to a later one), Q_10_ increased for the grassland clay and silt fractions (Table 2).

**Table 2:** The Q_10_ * values (±SD) of SOC decomposition for different particle-size fractions in grassland and forest soils (n = 4).

Land uses	Temperature-change cycles	Particle size fractions		
		Clay (<2 µm)	Silt (2−50 µm)	Sand (>50 µm)
Grassland	1	2.56±0.07a¶	2.41±0.05b	2.39±0.04b
	2	2.60±0.04a	2.45±0.06b	2.34±0.09c
	3	2.88±0.04a	2.51±0.02b	2.27±0.07c
	Mean§	2.68±0.16aA#	2.45±0.06bA	2.33±0.08cA
Forest	1	2.45±0.07a	2.32±0.08b	2.19±0.04c
	2	2.24±0.01a	2.13±0.08b	2.08±0.03b
	3	2.59±0.04a	2.23±0.05b	2.00±0.05c
	Mean§	2.43±0.16aB	2.22±0.10bB	2.09±0.09cB

*Q_10_ value was calculated using Eqn. 3 with the parameters for the fitted curves in Figure 2.

§ The mean was calculated including each replicate among the three temperature-change cycles (n = 12).

¶ Different lowercase letters in the same row indicate significance differences (*p*<0.05) among soil fractions.

# Different capital letters in the same column indicate significance differences (*p*<0.05) between two land uses.

**Figure 3:**
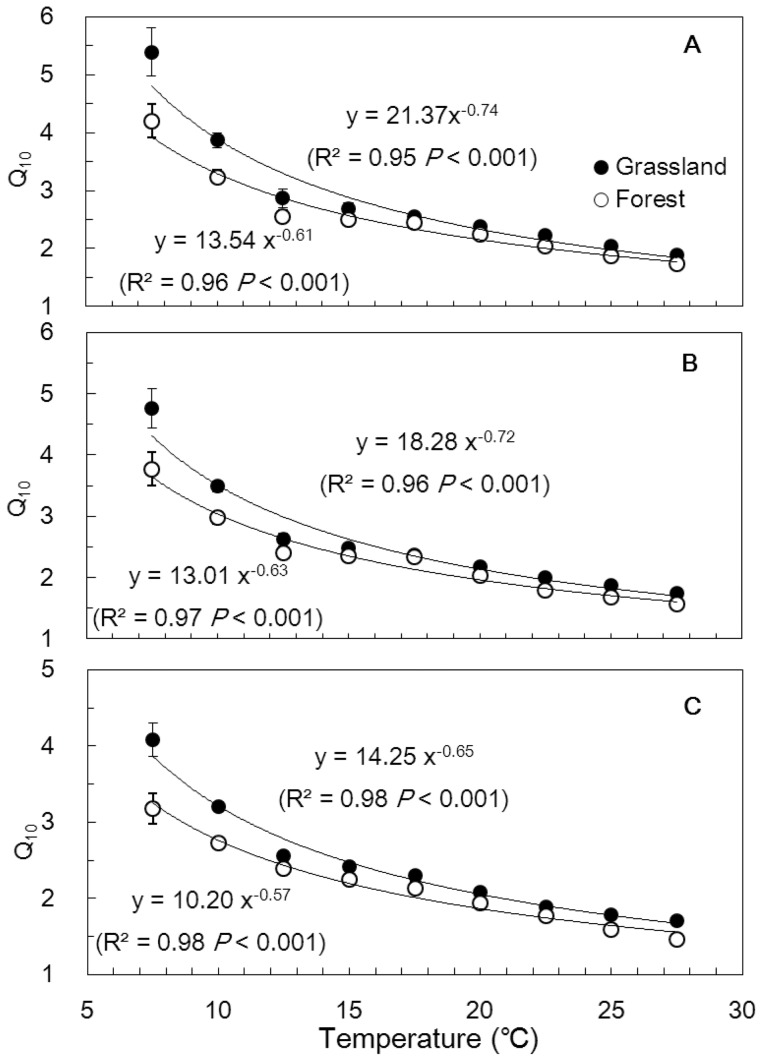
Dependence of Q10 on incubation temperature for (A) clay, (B) silt, and (C) sand. The Q10 for each temperature interval of 5 degrees (e.g., 5–10°C) and 10 degrees (e.g., 5–15°C) was calculated using Eqn. 4. The average temperature for each interval was taken as representative temperature. Data from all the three temperature-change cycles were included. Vertical bars denote the standard errors (n = 12).

The Q_10_ values decreased in power-law with increasing temperature for all soil particles when calculating using Eqn. 4 during each individual temperature interval (Figure 3). The average Q_10_ values of all soil particle fractions were 4.22±0.14 during the minimum temperature interval and 1.81±0.17 during the maximum temperature interval (Figure 3). The grassland soil particles had greater Q_10_ than the corresponding forest soil particles during the temperature interval of 5–15°C, whereas the Q_10_ values for the two land uses tended to approach one other during 15–30°C (Figure 3). Nevertheless, the grassland soil particles exhibited a more rapid decline in Q_10_ with increasing temperature (i.e., greater absolute values of the power-law exponents in Figure 3) than did the forest soil particles. Furthermore, the Q_10_ values calculated using Eqn. 4 followed as clay>silt>sand (Figure 3), which was consistent with the pattern in Q_10_ calculated using the exponential model (Eqn. 3) (Table 2).

### Dependence of Q_10_ on soil biochemical properties

Generally, there were large differences among various particle-size fractions in most soil biological and chemical properties at the end of incubation (Table 3). Specifically, MBC, MBN, DOC, and TDN were greatest in the clay fraction and were generally of the same order of magnitude as Q_10_ (Table 2). However, the ratios of MBC/OC and MBN/TN shifted to be greatest in the sand fraction. The microbial C/N ratios were not significantly different among the soil particles, but they were generally greater than the soil particles' C/N ratios (except for the forest sand particles; see Table 1). Additionally, the grassland soil particles had greater MBC/OC and less qCO_2_ values than the corresponding forest soil particles.

**Table 3:** Soil biological and chemical properties in different particle-size fractions at the end of incubation (mean±SE, n = 4).

Land uses and soil fractions	MBC (mg/kg)	MBN (mg/kg)	DOC (mg/kg)	TDN (mg/kg)	MBC/OC*(‰)	MBN/TN* (‰)	Micro C/N (kg/kg)	qCO_2_ § (mg CO_2_-C kg^−1^ MBC h^−1^)
Grassland								
Clay (<2 µm)	1096±43a¶	35±7a	236±21a	552±3a	20±1c	6±1b	36±7a	480±25a
Silt (2−50 µm)	831±22b	29±4a	65±4b	179±1b	27±1b	9±1b	29±3a	400±14b
Sand (>50 µm)	457±16c	22±1a	38±4b	39±1c	51±2a	33±2a	21±2a	531±14a
Forest								
Clay (<2 µm)	515±46a	61±24a	262±17a	372±21a	7±1b	10±4a	17±8a	896±72b
Silt (2−50 µm)	188±7b	11±0b	114±9b	65±1b	11±0a	8±0a	17±1a	708±32c
Sand (>50 µm)	169±15b	13±3b	122±6b	31±2b	13±1a	19±5a	16±3a	1117±93a

*MBC/OC and MBN/TN refer to the ratio of MBC to fraction-OC and of MBN to fraction-TN, respectively.

§ qCO_2_ is the metabolic quotient calculated by the CO_2_ emission rate at 25°C in the third incubation cycle divided by MBC.

¶ Different lowercase letters in the same column for the same land use indicate significance differences (*p*<0.05) among soil fractions.

**Figure 4:**
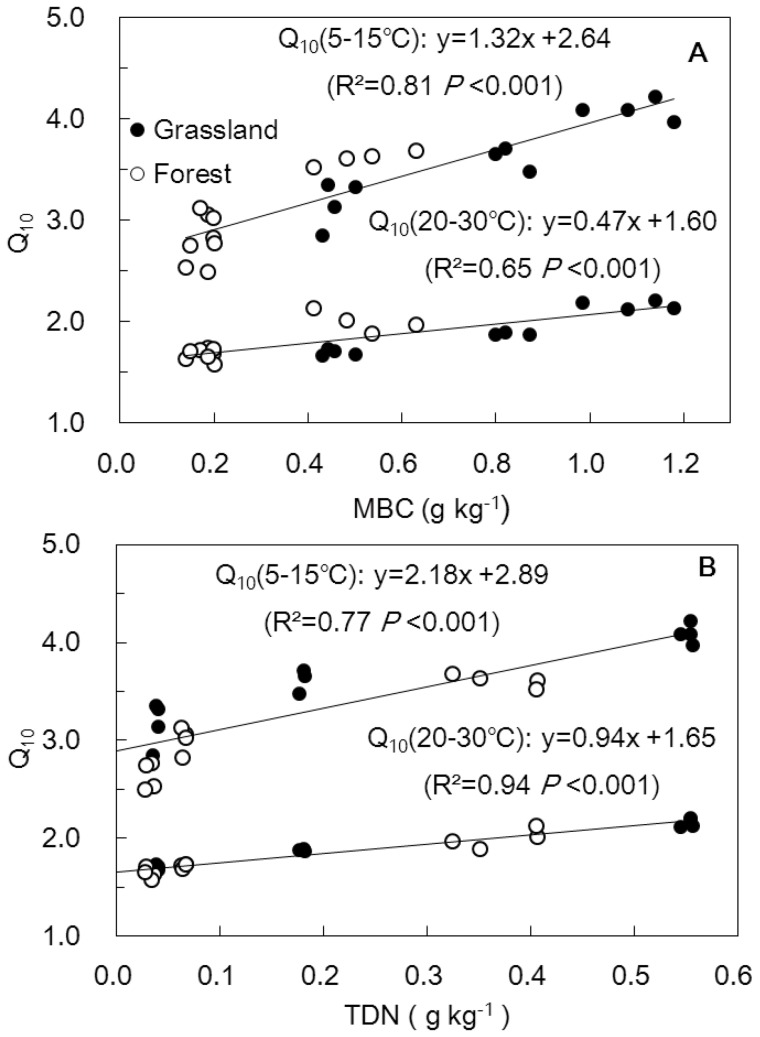
Dependence of Q10 in the third temperature-change cycle on microbial biomass carbon (A) and total dissolved nitrogen (B). The Q10 (5−15°C) and Q10 (20−30°C) were calculated using Eqn. 4 for each replicate.

The soil biological and chemical properties at the end of incubation represented the soil conditions during the 3^rd^ temperature-change cycle; thus we analyzed the relationship between the Q_10_ in the 3^rd^ cycle and these soil biological and chemical properties. Table 4 presents the Pearson correlation coefficients of Q_10_ during an intermediate temperature range of 15–25°C with various soil biochemical properties. Clearly, the Q_10_ values were highly correlated with MBC and TDN. The Q_10_ had significant linear relationships with MBC (Figure 4A) and TDN (Figure 4B) for the 5–15°C and 20–30°C temperature intervals and also for other temperature intervals (not shown). It should be noted that the slopes of regression line were different between the temperature ranges of 5–15°C and 20–30°C. Over an increasing temperature interval of 10°C, the slopes with low baseline levels of 5°C were steeper than those with high baseline levels of 20°C. Note that the datasets were well-mixed for the grassland and forest, thereby suggesting that these two land use soils had similar features in terms of the dependence of Q_10_ on MBC and TDN.

**Table 4:** Pearson correlation coefficient for Q_10_ (15–25°C) in the third temperature-change cycle and soil biological and chemical properties.

	Q_10_ (15–25°C) a	MBC	MBN	DOC	TDN	Micro C/N	DOC/TDN	MBC/OC	MBN/TN	qCO_2_
Q_10_ (15–25°C)	1									
MBC	0.87^***^	1								
MBN	0.52*	0.44*	1							
DOC	0.58^**^	0.32	0.38	1						
TDN	0.90^***^	0.80^***^	0.45*	0.81^***^	1					
Micro C/N	0.51*	0.59^**^	−0.29	0.25	0.54^**^	1				
DOC/TDN	−0.85^***^	−0.73^***^	−0.42	−0.20	−0.59^**^	−0.45*	1			
MBC/OC	−0.02	0.19	−0.09	0.65^**^	0.29	0.13	0.19	1		
MBN/TN	−0.43*	−0.29	0.04	−0.59^**^	−0.54^**^	−0.44*	0.19	0.71^***^	1	
qCO_2_	−0.66^***^	−0.70^***^	−0.24	0.18	−0.35	−0.37	0.85^***^	−0.54*	−0.05	1

a Q_10_ (15−25°C) was calculated using Eqn. 4 for each replicate.

*, **, ***Significant at probability levels of 0.05, 0.01, and 0.001, respectively.

## Discussion

### Carbon stability and microbial activity among different size fractions

The carbon associated with different particle-size fractions has different availability and lability [42]. The sand fraction had greater organic carbon availability and lability (greater parameter A), and thus lower carbon stability, than did the silt and clay fractions (Fig. 2). This pattern of SOC stability among the different size fractions was consistent with previous studies [43], [44]. The difference in SOC stability among soil primary particles is the reason why coarse sandy soils that are rich in sand particles are less stable and exhibit faster SOC turnover rates than fine-textured soils that are rich in clay and silt particles [45].

Land-use type can affect the SOC stability by inputting carbon into the soil via litter or roots with different chemical recalcitrance [18], [46]. The sand fraction had greater carbon stability (smaller parameter A) in the forest than in the grassland soil (Fig. 2). A possible reason is that the organic matter in the sand fraction is primarily composed of partially decomposed plant litter [18]; forest litter has higher lignin concentration and higher C:N ratio than grassland litter and is thus usually more recalcitrant [47]. In contrast, the carbon stability (parameter A) in clay or silt fractions was similar between grassland and forest during each temperature-change cycle (Fig. 2), thereby suggesting that the organic carbon stabilities in clay and silt fractions were not affected by land-cover type. The carbon stability in these fine fractions is primarily related to organo-mineral association (i.e., chemical protection) [48]. Overall, the SOC stability in the sand fraction is primarily determined by the chemical recalcitrance of organic matter but is more strongly determined by the association of SOC with minerals in the clay or silt fractions. This pattern was consistent with different determinants of organic matter-derived nitrogen preservation between the sandy soils and clay soils [49].

Microbial activities and distribution may differ among different size fractions because these fractions provide distinct microhabitats and various organic carbon qualities and quantities [50]–[52]. Clay fraction had the greatest organic carbon concentration (Table 1) and had the maximum amount of MBC (Table 3), thereby indicating that the greatest biological activity was in clay fractions [18]. However, the clay fraction had the least MBC/OC ratio (Table 3), an eco-physiological parameter that refers to the microbial efficiency in using carbon substrate [53]. The microbes in the clay fraction had the lowest carbon use efficiency, which is likely related to low carbon availability due to strong organo-mineral association. Moreover, the grassland soil particles had greater MBC/OC and lower qCO_2_, i.e., microbial community respiration per unit microbial biomass [53], than the corresponding forest particles (Table 3), thereby suggesting that the grassland soil contains a microbial community that is more efficient at using carbon energy.

### Effect of climatic and soil parameters on Q_10_

**Figure 5:**
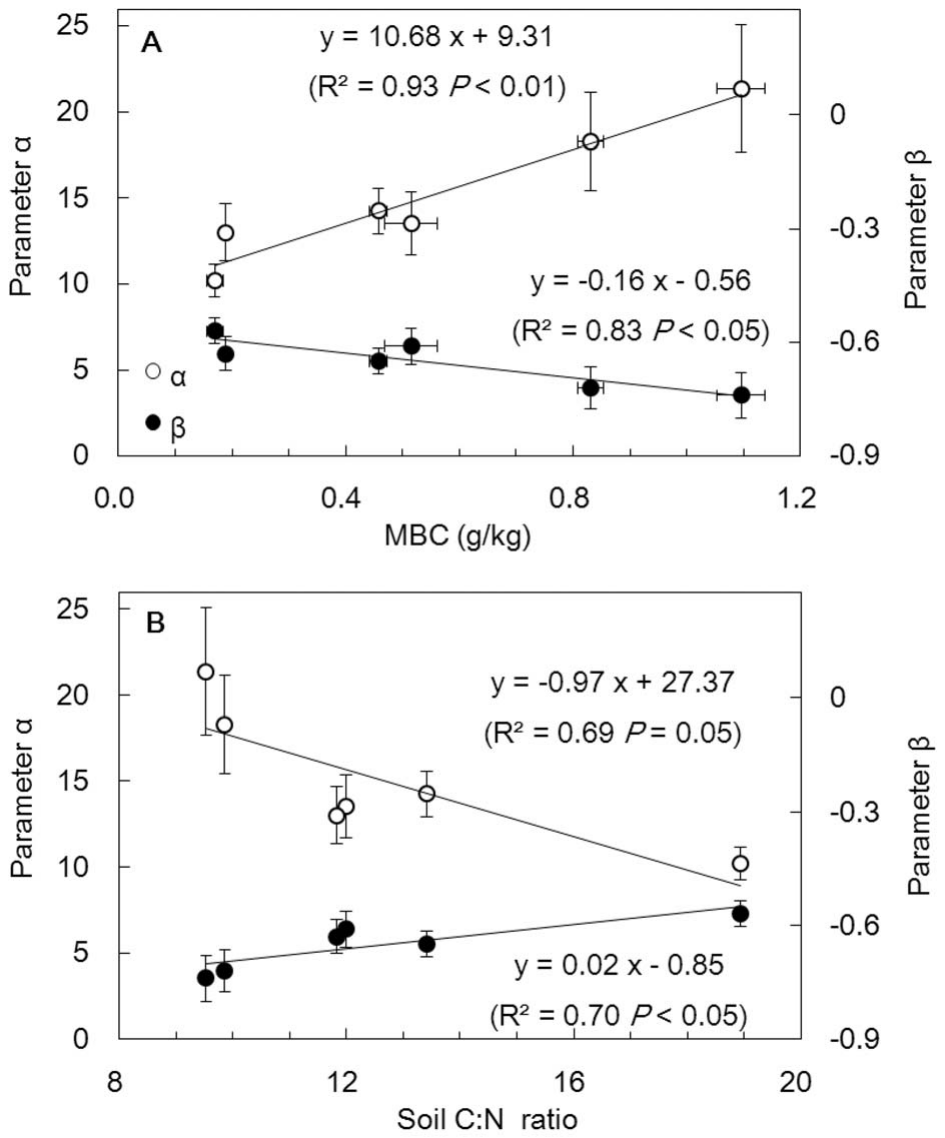
Dependence of parameters in the Q10−T curve on the soil microbial biomass carbon (A) and soil C: N ratio (B). Vertical bars denote the 95% CI of the estimated parameters in Fig. 3. For each parameter α and β, six points represent the three particles of clay, silt, and sand from the grassland and forest soils. Horizontal bars represent the standard errors (n = 4).

First, temperature can determine the Q_10_ of SOC decomposition. The lower Q_10_ of soil respiration at higher temperatures was not only assumed by the Arrhenius equation [6] but also confirmed by two comprehensive analyses of incubation studies of soils from all types of terrestrial ecosystems [11], [54]. Similarly, we found that the Q_10_ values were negatively correlated with the incubation temperature (Figure 3). The relationship was fitted by a power function: 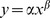. Clearly, higher values of α produce higher values of Q_10_. Otherwise, with larger absolute values of β, Q_10_ decreases more rapidly with increasing temperature. The parameters α and β in the Q_10_-T curves were found to be significantly correlated with the MBC and soil C:N ratio (Figure 5). An increase in the MBC concentration would lead to α and Q_10_ rising (Figure 5A), which is consistent with Figure 4A. Significant inverse relationships were found between the parameter α and the soil C:N ratio (Figure 5B), thereby suggesting that higher soil C:N ratios was linked with higher tolerance to increasing temperature. A more rapid rate of Q_10_ decrease with increasing temperature (greater absolute value of β) in grassland versus forest soil particles could be related to lower C:N ratios (Table 1) or greater MBC in grassland soil particles (Table 3). It should be mentioned that no significant relationship was found between the values of α and β and the soil properties of MBN, DOC, and DTN.

The negative relationship between Q_10_ of SOC decomposition and temperature has two implications. First, in a given region, SOC decomposition should be more sensitive to warming during winter than warming during summer. This trend was observed in the Alaskan tundra, with an increase of greater than 50% in CO_2_ fluxes during the snow-covered period by winter warming and a 20% increase during the growing season by summer warming [55]. Nevertheless, considering that the initial soil respiration rate is much less in winter than in summer, the absolute magnitude of the CO_2_ emission increase from warming could be still less in winter than in summer. Second, the SOC stock in boreal and temperate regions (low MAT), especially peat lands and permafrost, should be more vulnerable to warming than that in subtropical and tropical regions (high MAT) [6]. Moreover, peat lands and permafrost contain more than 50% of global soil carbon stock [56] and have been experiencing remarkable warming [57]. Consequently, peat lands and permafrost are expected to experience largest potential soil carbon loss by 2100 due to global warming [6].

Second, soil texture could have an impact on Q_10_ of SOC decomposition. The Q_10_ decreased in the order clay>silt>sand seemingly regardless of land-cover types, soil types, and climatic factors (Table 2). Consequently, SOC decomposition in fine-textured soils (rich in clay and silt content) could be more sensitive to global warming than that in coarse-textured soils (rich in sand content). Moreover, fine-textured soils generally have greater capacities to preserve SOC [58] and thus contain more SOC than do coarse-textured soils [1], [59]. Therefore, current global model predictions that use constant Q_10_ values for different soil textures may predict an underestimated response of the soil carbon cycle to climate warming.

Third, the soil nitrogen availability, indicated by TDN, could also affect the Q_10_ values, and greater Q_10_ values were related to higher TDN content regardless of land use type (Figure 4B). This result agrees with other incubation studies: Chang et al. [60] demonstrated the existence of a positive relationship between Q_10_ and the soil total nitrogen content, and Cusack et al. [61] observed that nitrogen fertilization significantly increased the Q_10_ of slow SOC pools. This relationship between Q_10_ and nitrogen availability could result from the nitrogen availability driving the activities of the soil enzymes that acquire carbon [62]. Low nitrogen availability may inhibit enzyme activity and thus constrain the warming response of SOC decomposition. This close relation of Q_10_ with soil nitrogen availability suggests that atmospheric nitrogen deposition could stimulate the positive feedback of soil respiration to climate warming.

### Q_10_ for labile carbon pools vs. Q_10_ for recalcitrant carbon pools

The question of whether the decomposition of recalcitrant and labile carbon has different temperature dependencies is important because most experimental work has been done on the temperature sensitivity of the decomposition of more labile fractions, yet the long-term effect of warming on soil carbon storage should be primarily determined by the temperature dependence of the more recalcitrant carbon pool [7]. Kinetic theory and the Arrhenius equation both suppose that recalcitrant carbon pools are more warming-sensitive than labile carbon pools [6], [63]. Many laboratory incubation studies have supported this supposition [32], [64], [65]. Our study further affirmed this result by showing that the decomposition of the carbon associated with the clay and silt fractions (recalcitrant carbon pool), with slower turnover rates (Figure 2), yet had higher Q_10_ than that with the sand fraction (labile carbon pool) (Table 2). Furthermore, the organic carbon associated with the clay and silt fractions usually accounts for a large portion of the total SOC, i.e., more than 60% in this study (Table 1). Therefore, long-term response of the SOC stock to climate warming would be more severe than the current predictions that assume the same Q_10_ for labile and recalcitrant carbon pools.

However, the supposition that recalcitrant carbon pools have greater warming dependence has not obtained wide acceptance [31], [66]. Based on this supposition, Q_10_ in the incubation studies is expected to rise with prolonged incubation time because SOC is generally composed of a large proportion of labile carbon during the early incubation period, yet of a small proportion of labile carbon during the late incubation period due to depletion of labile carbon [67]. And yet, Fang et al. [31] and Reichstein et al. [66] found that Q_10_ was stable and independent of the incubation time. Similarly, our study only observed increasing trends in Q_10_ for the grassland clay and silt fractions from the 1^st^ temperature-change cycle to the 2^nd^ and the 3^rd^ (Table 2) and did not found such trends in Q_10_ for the forest soil fractions or the grassland sand fractions. The inconsistency between these observations and the expectation derived from the above supposition could be explained by that Q_10_ having a significant positive relation with MBC and lower MBC values resulting in a smaller Q_10_ (Figure 4A); MBC generally declines with prolonged incubation because of the depletion of labile carbon [31], [68]. Consequently, as incubation is prolonged, increasing trend of Q_10_ due to increased recalcitrant carbon proportion could be counteracted by decreasing trend of Q_10_ as a result of declined MBC [67]. If the MBC did not decrease sharply from early to late incubation stage, the Q_10_ would have increased as incubation progressed in Fang et al. [31] and Reichstein et al. [66], as well as in our study. However, the mechanism for how Q_10_ is affected by MBC is not clear. Assuming that the microbial community composition does not change and that every single microbe maintains the same warming response with prolonged incubation time, a decline in MBC alone should not induce lower Q_10_. Possible mechanisms could be either microbial community shift to species with low thermal response [69] or microbe acclimatization to temperature increase [70]. On the other hand, soil carbon recalcitrance did not certainly increase continuously with prolonged incubation. In other words, carbon availability and lability did not certainly decease continuously. Such as, we can see that the parameter A for the sand fraction increased from the second to the third cycle both for grassland and forest soils (Figure 2). Tian et al. [71] also observed the phenomenon and affirmed that a pulse-dynamic pattern of soil respiration and increased carbon availability during a long incubation could exist. Overall, these results suggest that it is unreliable to judge whether the decomposition of recalcitrant and labile carbon have different temperature dependencies by comparing Q_10_ values after different incubation time.

## Conclusions

This study presented three implications for understanding the soil-carbon response to climate warming. First, the Q_10_ of SOC decomposition in soil particles decreased with increasing particle size (clay>silt>sand) for grassland and forest soils. This result suggested that SOC decomposition from fine-textured soils rich in clay or silt content could be more sensitive to warming than coarse sandy soils. Second, Q_10_ decreased with increasing temperature, thereby implicating that the SOC might be more vulnerable in low MAT regions than in high MAT regions under future warming. Third, the soil nitrogen availability facilitated a temperature dependence of SOC decomposition, suggesting that atmospheric nitrogen deposition could stimulate positive feedback of soil respiration on climate warming.

## References

[1] Jobbagy E, Jackson R (2000) The vertical distribution of soil organic carbon and its relation to climate and vegtation. Ecol Appl 10: 423–436.

[2] Cox PM, Betts RA, Jones CD, Spall SA, Totterdell IJ (2000) Acceleration of global warming due to carbon-cycle feedbacks in a coupled climate model. Nature 408: 184–187. 11089968 10.1038/35041539

[3] Bond-Lamberty B, Thomson A (2010) Temperature-associated increases in the global soil respiration record. Nature 464: 579–582. 20336143 10.1038/nature08930

[4] Schimel DS, Braswell BH, Holland EA, McKeown R, Ojima DS, et al (1994) Climatic, edaphic, and biotic controls over storage and turnover of carbon in soils. Global Biogeochem Cy 8: 279–293.

[5] Lloyd J, Taylor JA (1994) On the temperature dependence of soil respiration. Funct Ecol 8: 315–323.

[6] Davidson EA, Janssens IA (2006) Temperature sensitivity of soil carbon decomposition and feedbacks to climate change. Nature 440: 165–173. 16525463 10.1038/nature04514

[7] Kirschbaum MUF (2006) The temperature dependence of organic-matter decomposition—still a topic of debate. Soil Biol Biochem 38: 2510–2518.

[8] Friedlingstein P, Cox P, Betts R, Bopp L, Von Bloh W, et al (2006) Climate-carbon cycle feedback analysis: Results from the C(4)MIP model intercomparison. J Climate 19: 3337–3353.

[9] Zhou T, Shi P, Hui D, Luo Y (2009) Global pattern of temperature sensitivity of soil heterotrophic respiration (Q_10_) and its implications for carbon-climate feedback. J Geophys Res 114: , G02016.

[10] Raich JW, Schlesinger WH (1992) The global carbon-dioxide flux in soil respiration and its relationship to vegetation and climate. Tellus B 44: 81–99.

[11] Kirschbaum MUF (1995) The temperature-dependence of soil organic-matter decomposition, and the effect of global warming on soil organic-C storage. Soil Biol Biochem 27: 753–760.

[12] Davidson EA, Belk E, Boone RD (1998) Soil water content and temperature as independent or confounded factors controlling soil respiration in a temperate mixed hardwood forest. Global Change Biol 4: 217–227.

[13] Suseela V, Conant RT, Wallenstein MD, Dukes JS (2012) Effects of soil moisture on the temperature sensitivity of heterotrophic respiration vary seasonally in an old-field climate change experiment. Global Change Biol 18: 336–348.

[14] Wan S, Luo Y (2003) Substrate regulation of soil respiration in a tallgrass prairie: results of a clipping and shading experiment. Global Biogeochem Cy 17: , 1054.

[15] Fierer N, Colman BP, Schimel JP, Jackson RB (2006) Predicting the temperature dependence of microbial respiration in soil: a continental-scale analysis. Global Biogeochem Cy 20: , GB3026.

[16] Dieckow J, Bayer C, Conceicao PC, Zanatta JA, Martin-Neto L, et al (2009) Land use, tillage, texture and organic matter stock and composition in tropical and subtropical Brazilian soils. Eur J Soil Sci 60: 240–249.

[17] Zinn YL, Lal R, Resck DVS (2005) Changes in soil organic carbon stocks under agriculture in Brazil. Soil Till Res 84: 28–40.

[18] Christensen BT (2001) Physical fractionation of soil and structural and functional complexity in organic matter turnover. Eur J Soil Sci 52: 345–353.

[19] Sposito G, Skipper NT, Sutton R, Park S-h, Soper AK, et al (1999) Surface geochemistry of the clay minerals. P Natl Acad Sci USA 96: 3358–3364. 10.1073/pnas.96.7.3358 PMC34275 10097044

[20] von Lützow M, Kögel-Knabner I, Ekschmitt K, Flessa H, Guggenberger G, et al (2007) SOM fractionation methods: Relevance to functional pools and to stabilization mechanisms. Soil Biol Biochem 39: 2183–2207.

[21] Six J, Conant RT, Paul EA, Paustian K (2002) Stabilization mechanisms of soil organic matter: Implications for C-saturation of soils. Plant Soil 241: 155–176.

[22] Baldock JA, Skjemstad JO (2000) Role of the soil matrix and minerals in protecting natural organic materials against biological attack. Org Geochem 31: 697–710.

[23] Arevalo CBM, Chang SX, Bhatti JS, Sidders D (2012) Mineralization potential and temperature sensitivity of soil organic carbon under different land uses in the parkland region of Alberta, Canada. Soil Sci Soc Am J 76: 241–251.

[24] Feng W, Plante AF, Six J (2013) Improving estimates of maximal organic carbon stabilization by fine soil particles. Biogeochem 112: 1–13.

[25] Yang XM, Drury CF, Reynolds WD, MacTavish DC (2009) Use of sonication to determine the size distributions of soil particles and organic matter. Can J Soil Sci 89: 413–419.

[26] Jackson ML (1969) Soil chemical analysis-advanced course. Wisconsin: University of Wisconsin. pp. 20–65.

[27] Tiessen H, Stewart JWB (1983) Particle-size fractions and their use in studies of soil organic-matter. 2. cultivation effects on organic-matter composition in size fractions. Soil Sci Soc Am J 47: 509–514.

[28] Christensen BT (1987) Decomposability of organic-matter in particle-size fractions from field soils with straw incorporation. Soil Biol Biochem 19: 429–435.

[29] Linn DM, Doran JW (1984) Effect of water-filled pore-space on carbon-dioxide and nitrous-oxide production in tilled and nontilled soils. Soil Sci Soc Am J 48: 1267–1272.

[30] Chen X, Tang J, Jiang L, Li B, Chen J, et al (2010) Evaluating the impacts of incubation procedures on estimated Q_10_ values of soil respiration. Soil Biol Biochem 42: 2282–2288.

[31] Fang CM, Smith P, Moncrieff JB, Smith JU (2005) Similar response of labile and resistant soil organic matter pools to changes in temperature. Nature 433: 57–59. 15635408 10.1038/nature03138

[32] Xu X, Luo YQ, Zhou JZ (2012) Carbon quality and the temperature sensitivity of soil organic carbon decomposition in a tallgrass prairie. Soil Biol Biochem 50: 142–148.

[33] Huang Y, Zou JW, Zheng XH, Wang YS, Xu XK (2004) Nitrous oxide emissions as influenced by amendment of plant residues with different C:N ratios. Soil Biol Biochem 36: 973–981.

[34] Bouyoucos GJ (1962) Hydrometer method improved for making particle size analyses of soils. Agron J 54: 464–465.

[35] Nelson DW, Sommers LE (1996) Total carbon, organic carbon, and organic matter. In: Sparks DL, Page AL, Helmke PA, Loeppert RH, Soltanpour PN, et al.., editors. Methods of soil analysis Part 3, chemical methods. Wisconsin: ASA, CSSA and SSSA. pp. 961–1010.

[36] Bremner J (1996) Nitrogen-total. In: Sparks DL, Page AL, Helmke PA, Loeppert RH, Soltanpour PN, et al.., editors. Methods of soil analysis Part 3, chemical methods. Wisconsin: ASA, CSSA and SSSA. pp. 1085–1121.

[37] Vance ED, Brookes PC, Jenkinson DS (1987) An extraction method for measuring soil microbial biomass-C. Soil Biol Biochem 19: 703–707.

[38] Jones DL, Willett VB (2006) Experimental evaluation of methods to quantify dissolved organic nitrogen (DON) and dissolved organic carbon (DOC) in soil. Soil Biol Biochem 38: 991–999.

[39] Li LJ, Zeng DH, Yu ZA, Fan ZP, Mao R (2010) Soil microbial properties under N and P additions in a semi-arid, sandy grassland. Biol Fert Soils 46: 653–658.

[40] Lang M, Cai Z, Chang SX (2011) Effects of land use type and incubation temperature on greenhouse gas emissions from Chinese and Canadian soils. J Soil Sediment 11: 15–24.

[41] Mikan CJ, Schimel JP, Doyle AP (2002) Temperature controls of microbial respiration in arctic tundra soils above and below freezing. Soil Biol Biochem 34: 1785–1795.

[42] Balesdent J, Mariotti A, Guillet B (1987) Natural ^13^C abundance as a tracer for studies of soil organic matter dynamics. Soil Biol Biochem 19: 25–30.

[43] Balesdent J, Mariotti A, (1996) Measurement of soil organic matter turnover using ^13^C natural abundance. In: Boutton T, Yamasaki S, editors. Mass spectrometry of soils. New York: Marcel Dekker. pp. 83–111.

[44] Ludwig B, John B, Ellerbrock R, Kaiser M, Flessa H (2003) Stabilization of carbon from maize in a sandy soil in a long-term experiment. Eur J Soil Sci 54: 117–126.

[45] Franzluebbers A (1999) Potential C and N mineralization and microbial biomass from intact and increasingly disturbed soils of varying texture. Soil Biol Biochem 31: 1083–1090.

[46] Chapman SK, Palanivel RU, Langley JA (2012) Soil carbon stability responds to land-use and groundcover management in southern appalachian agroecosystems. Soil Sci Soc Am J 76: 2221–2229.

[47] Kirschbaum MU, Guo LB, Gifford RM (2008) Why does rainfall affect the trend in soil carbon after converting pastures to forests?: A possible explanation based on nitrogen dynamics. Forest Ecol Manag 255: 2990–3000.

[48] Manjaiah KM, Kumar S, Sachdev MS, Sachdev P, Datta SC (2010) Study of clay-organic complexes. Curr Sci 98: 915–921.

[49] Chivenge P, Vanlauwe B, Gentile R, Six J (2011) Comparison of organic versus mineral resource effects on short-term aggregate carbon and nitrogen dynamics in a sandy soil versus a fine textured soil. Agr Ecosyst Environ 140: 361–371.

[50] Sessitsch A, Weilharter A, Gerzabek MH, Kirchmann H, Kandeler E (2001) Microbial population structures in soil particle size fractions of a long-term fertilizer field experiment. Appl Environ Microb 67: 4215–4224. 10.1128/AEM.67.9.4215-4224.2001 PMC93150 11526026

[51] Lagomarsino A, Grego S, Kandeler E (2012) Soil organic carbon distribution drives microbial activity and functional diversity in particle and aggregate-size fractions. Pedobiologia 55: 101–110.

[52] Kogel-Knabner I, Guggenberger G, Kleber M, Kandeler E, Kalbitz K, et al (2008) Organo-mineral associations in temperate soils: Integrating biology, mineralogy, and organic matter chemistry. J Plant Nutr Soil Sci 171: 61–82.

[53] Anderson TH, Domsch KH (2010) Soil microbial biomass: The eco-physiological approach. Soil Biol Biochem 42: 2039–2043.

[54] Hamdi S, Moyano F, Sall S, Bernoux M, Chevallier T (2013) Synthesis analysis of the temperature sensitivity of soil respiration from laboratory studies in relation to incubation methods and soil conditions. Soil Biol Biochem 58: 115–126.

[55] Natali SM, Schuur EAG, Trucco C, Pries CEH, Crummer KG, et al (2011) Effects of experimental warming of air, soil and permafrost on carbon balance in Alaskan tundra. Global Change Biol 17: 1394–1407.

[56] Tarnocai C, Canadell JG, Schuur EAG, Kuhry P, Mazhitova G, et al. (2009) Soil organic carbon pools in the northern circumpolar permafrost region. Global Biogeochem Cy 23: ,GB2023.

[57] Chapin FS, Sturm M, Serreze MC, McFadden JP, Key JR, et al (2005) Role of land-surface changes in Arctic summer warming. Science 310: 657–660. 16179434 10.1126/science.1117368

[58] Hassink J (1994) Effects of soil texture and grassland management on soil organic C and N and rates of C and N mineralization. Soil Biol Biochem 26: 1221–1231.

[59] Burke I, Yonker C, Parton W, Cole C, Schimel D, et al (1989) Texture, climate, and cultivation effects on soil organic matter content in US grassland soils. Soil Sci Soc Am J 53: 800–805.

[60] Chang XF, Zhu XX, Wang SP, Luo CY, Zhang ZH, et al (2012) Temperature and moisture effects on soil respiration in alpine grasslands. Soil Sci 177: 554–560.

[61] Cusack DF, Torn MS, McDowell WH, Silver WL (2010) The response of heterotrophic activity and carbon cycling to nitrogen additions and warming in two tropical soils. Global Change Biol 16: 2555–2572.

[62] Cusack DF (2013) Soil nitrogen levels are linked to decomposition enzyme activities along an urban-remote tropical forest gradient. Soil Biol Biochem 57: 192–203.

[63] Bosatta E, Agren GI (1999) Soil organic matter quality interpreted thermodynamically. Soil Biol Biochem 31: 1889–1891.

[64] Hartley IP, Ineson P (2008) Substrate quality and the temperature sensitivity of soil organic matter decomposition. Soil Biol Biochem 40: 1567–1574.

[65] Leifeld J, Fuhrer J (2005) The temperature response of CO_2_ production from bulk soils and soil fractions is related to soil organic matter quality. Biogeochem 75: 433–453.

[66] Reichstein M, Subke JA, Angeli AC, Tenhunen JD (2005) Does the temperature sensitivity of decomposition of soil organic matter depend upon water content, soil horizon, or incubation time? Global Change Biol 11: 1754–1767.

[67] Conant RT, Steinweg JM, Haddix ML, Paul EA, Plante AF, et al (2008) Experimental warming shows that decomposition temperature sensitivity increases with soil organic matter recalcitrance. Ecology 89: 2384–2391. 18831158 10.1890/08-0137.1

[68] Follett RF, Paul EA, Pruessner EG (2007) Soil carbon dynamics during a long-term incubation study involving C-13 and C-14 measurements. Soil Sci 172: 189–208.

[69] Zogg GP, Zak DR, Ringelberg DB, MacDonald NW, Pregitzer KS, et al (1997) Compositional and functional shifts in microbial communities due to soil warming. Soil Sci Soc Am J 61: 475–481.

[70] Luo YQ, Wan SQ, Hui DF, Wallace LL (2001) Acclimatization of soil respiration to warming in a tall grass prairie. Nature 413: 622–625. 11675783 10.1038/35098065

[71] Tian QX, He HB, Cheng WX, Zhang XD (2014) Pulse-dynamic and monotonic decline patterns of soil respiration in long term laboratory microcosms. Soil Biol Biochem 68: 329–336.

