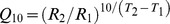# Correction: Decomposition of Organic Carbon in Fine Soil Particles Is Likely More Sensitive to Warming than in Coarse Particles: An Incubation Study with Temperate Grassland and Forest Soils in Northern China

**DOI:** 10.1371/journal.pone.0103801

**Published:** 2014-07-22

**Authors:** The PLOS ONE Staff

In the Materials and Methods section, there is an error in the fourth equation in the first set of parentheses. R_1_ should not be subtracted from R_2_; rather, R_2_ should be divided by R_1_. Please view the complete, correct equation here: